# Egg quality depending on the diet with different sources of protein and age of the hens

**DOI:** 10.1038/s41598-021-82313-1

**Published:** 2021-01-29

**Authors:** Emilia Kowalska, Joanna Kucharska-Gaca, Joanna Kuźniacka, Lidia Lewko, Ewa Gornowicz, Jakub Biesek, Marek Adamski

**Affiliations:** 1 Department of Animal Breeding and Nutrition, Faculty of Animal Breeding and Biology, UTP - University of Science and Technology in Bydgoszcz, Mazowiecka 28, 85-084 Bydgoszcz, Poland; 2 Water Poultry Genetic Resource Station in Dworzyska, Kołuda Wielka Experimental Station, Institute of Animal Production - National Research Institute, 32-065 Kórnik, Poland

**Keywords:** Fatty acids, Zoology

## Abstract

Due to the planned limitations in the use of genetically modified soybean meal, the search for alternative sources of protein in animal nutrition is ongoing, which also supports the consumers’ expectations of good quality products, such as eggs. The aim of the study was to assess and compare morphological traits of eggs, fatty acid composition in yolk lipids, and the content and activity of lysozyme in thick and thin albumen in eggs from hens fed a diet based on legume seeds as a substitute for soybean meal depending on the hens age. Analyses were carried out for 300 eggs on dates I–V (19–39 weeks age of hens), obtained from Rosa 1 hens managed in the semi-intensive system. Quality analysis was performed for 30 eggs from each group at a time. The control group of hens (A) was fed a diet based on soybean meal (SBM) and the treatment group (B) was fed a diet based on seeds from narrow-leaved lupin (Boruta), yellow lupin (Mister), and pea (Muza). Eggs were analysed for morphological traits (egg weight, the weight and density of egg components, egg shape index and egg surface area), parameters of albumen (height, Haugh units), yolk colour (La Roche, colorimetry in the CIE Lab system), lysozyme content and activity in albumen, and fatty acids composition in yolk lipids. In group B the egg shape index was higher (*p* = 0.002), and the eggshell colour index was lower (*p* = 0.007), as well as the height of thick albumen, Haugh score, and yolk colour (La Roche) were higher in group B (*p* < 0.05), while redness of yolk was significantly lower (*p* < 0.001) than in group A. Lower content of C15:0 and C18:1n9 and higher content of C18:2n6 and C18:3n3, (*p* < 0.05), as well as lower content of MUFA and OMEGA 9, but higher content of PUFA, OMEGA 3, OMEGA 6, PUFA/SFA, OMEGA 9/6 and OMEGA 9/3 were found in group B compared to group A (*p* < 0.05). There were significant differences in traits between age of hens (*p* < 0.05). Egg weight, yolk weight and its proportion in egg, as well as shell weight, its strength and thickness increased with the age of hens (*p* < 0.05). The content of lysozyme and its activity were also higher in eggs from older hens (*p* < 0.05). Fatty acids composition was beneficial at the beginning and end of the analysed egg production period. The proposed feed mixture based on legume seeds had no negative effect on the quality of eggs, and had a positive effect on yolk colour, the quality of albumen. There was no detrimental effect of diet and age of hens on fatty acid composition of eggs from both groups throughout the study period. To sum up, diet with legume seeds could be accepted as a substitute for soybean meal, due to the beneficial effects for some mentioned traits of eggs. Use of legumes could give a partial self-sufficient for producers from small farms, where is not possible to produce soybean meal. At the same time, the level of anti-nutrients in legumes should be considered.

## Introduction

The lack of environmental conditions suitable for the cultivation of soybean and production of soybean meal (SBM) has stimulated a growing interest in the use of legume seed in poultry diets^1^. Legume seeds may be an alternative on small farms which often produce feeds from their own crops^2^. Another factor driving research into alternative sources of protein is the constantly growing price of SBM^3^, especially if we consider obstacles to trade between countries associated with the 2020 pandemic^4^. Reportedly, lupins can be a real alternative to SBM without causing a negative effect on the quantity and quality of poultry products, such as eggs, as well as a meat from chickens, turkeys or waterfowl^5^.

Compared to SBM, legume seeds contain similar amounts of lysine and methionine, which are necessary in poultry diets^6^. Narrow-leaved (blue) lupin and yellow lupin are also known as sweet lupins, and they are rich in protein, and low in fat and starch^7^. According to Konieczka and Smulikowska^8^, lupin seeds (from various species) contain from 330 to 430 g of crude protein and 105 g crude fat per kg of dry weight. Peas are rich in protein and energy, and their amino acids composition is similar to that of SBM^9^. In the past, legumes were not recommended in the diet of monogastric animals due to the high content of antinutrients, such a tannins, protease inhibitors, oligosaccharides, or phytate and alkaloids, but today new varieties contain much lower levels of these substances, and there are no limitations to their use for the production of feed^10–13^.

Egg quality is determined based on many traits important for global egg production, and depends on many factors, including the diet and age of hens^14^. Previous studies indicated the possibility of using peas in the diet of laying hens with no negative impact on the quality of eggs^15^. Laudadio and Tufarelli^10^ reported that processed (dehulled-micronized) yellow lupin (at the level 18%) is a satisfactory component of hen diet and can be used instead of SBM, while Rutkowski et al*.*^11^ found that a graded inclusion of yellow lupin (max. 20%) had no negative effect on egg production or on some quality traits of hen eggs, such a shape index, percentage share of yolk and thick albumen content, shell quality, and beneficial effect on the yolk colour. On the other hand, researchers investigating the use of blue lupin in the diet of laying hens recommended the supplementation of feed with methionine, because unsupplemented blue lupin deteriorated egg’ albumen dry matter^16^. Another study revealed that the inclusion of narrow-leaved lupin (150 g/kg dry matter) had no negative effect on egg production, but the diet should be supplemented with enzymes digesting NSP to minimize the inclusion of SBM^17^.

The following research hypothesis was put forward: A diet based on peas (var. Muza), yellow lupin (var. Mister) and narrow-leaved lupin (var. Boruta) as alternatives to soybean meal (var. Hipro) used for Rosa 1 laying hens of different ages managed in the semi-intensive barn system influences morphological traits of eggs, physicochemical parameters of egg components (yolk, albumen, shell), fatty acids composition of yolk, and the content and activity of lysozyme in albumen. The aim of the research was to analyse the parameters mentioned in the research hypothesis.

## Results

### Effect of diet on egg quality

Results from the analysis of egg weight and surface area are presented in Table 1. The egg shape index was significantly higher in the treatment group (B) than in the control group (A) (*p* = 0.002). Whiter eggshells (shell colour) were found in group A (*p* = 0.007) (Table 2). Albumen from hens fed a diet based on legume seeds (B) was characterised by significantly greater height of thick albumen (*p* = 0.036), as well as significantly higher Haugh unit (HU) (*p* = 0.023), as presented in Table 3. Analysis of yolk colour (Table 4) revealed significantly higher values of DSM score in group B (*p* = 0.009), and significantly lower redness of yolk in group B compared to group A (*p* = 0.000).

**Table 1:** Mean values (x̅) and standard deviations (± SD) for the egg weight, egg shape index and egg surface area.

Factor	Indicator	Egg weight (g)	Egg shape index (%)	Egg surface area (cm^2^)
Group^1^	A	53.45	74.29^b^	67.19
	B	53.07	75.41^a^	66.84
	± SD*	± 6.41	± 3.22	± 5.37
	*p* value	0.441	0.002	0.291
Age of hens (weeks)	I (19)	44.54^d^	75.31^ab^	59.67^d^
	II (24)	52.05^c^	75.79^a^	66.00^c^
	III (29)	53.31^c^	73.70^b^	67.18^c^
	IV (34)	55.57^b^	75.14^ab^	69.73^b^
	V (39)	59.66^a^	74.31^b^	72.40^a^
	SD	3.86	3.15	3.24
	*p* value	0.000	0.000	0.000
	Interaction	0.717	0.121	0.650

Different letters (a, b) indicate a significant difference between treatments (A–B) and periods (I–V) at *p* < 0.05.

*Standard deviation.

^1^TREATMENT: A, control with soybean meal; B, experimental with yellow lupin, narrow-leaved lupin and peas.

**Table 2:** Mean values (x̅) and standard deviations (± SD) of the eggshell parameters.

Factor	Indicator	Shell colour (%)	Shell strength (g/cm^2^)	Shell weight (g)	Shell proportion in the egg (%)	Shell thickness (mm)	Shell density (g/cm^3^)
Group^1^	A	56.49^a^	3.92	4.90	9.21	0.309	2.063
	B	54.39^b^	3.86	4.84	9.16	0.309	2.050
	± SD*	± 7.11	± 0.83	± 0.61	± 0.90	± 0.03	± 0.01
	p value	0.007	0.488	0.182	0.669	0.903	0.266
Age of hens (weeks)	I (19)	52.34^c^	3.42^b^	4.13^d^	9.31	0.292^b^	2.036^b^
	II (24)	52.83^c^	4.07^a^	4.75^d^	9.19	0.309^b^	2.050^b^
	III (29)	56.07^b^	3.89^a^	4.84^c^	9.12	0.306^b^	2.070^b^
	IV (34)	58.14^a^	3.97^a^	5.16^b^	9.19	0.312^a^	2.085^a^
	V (39)	58.80^a^	4.11^a^	5.44^a^	9.12	0.319^a^	2.089^a^
	SD	6.76	2.08	1.43	0.89	0.02	0.10
	p value	0.000	0.000	0.000	0.795	0.000	0.023
	Interaction	0.825	0.036	0.316	0.215	0.475	0.245

Different letters (a, b) indicate a significant difference between treatments (A–B) and periods (I–V) at *p* < 0.05.

*Standard deviation.

^1^TREATMENT: A, control with soybean meal; B, experimental with yellow lupin, narrow-leaved lupin and peas.

**Table 3:** Mean values (x̅) and standard deviations (± SD) for the parameters of egg components.

Factor	Indicator	Weight (g)				Proportion in egg (%)				Height of thick albumen (mm)	Haugh units (HU)	Density	
		Thick albumen	Thin albumen	Total albumen	Yolk	Thick albumen	Thin albumen	Total albumen	Yolk			Thick albumen	Yolk
Group^1^	A	21.20	14.37	35.57	12.96	39.78	26.94	66.72	24.07	9.42^b^	97.59^b^	1.046	1.034
	B	20.93	14.24	35.24	12.95	39.70	26.95	66.65	24.18	9.81^a^	99.50^a^	1.048	1.028
	± SD*	± 3.43	± 3.48	± 4.07	± 2.78	± 5.01	± 6.02	± 3.41	± 3.25	± 2.10	± 10.09	± 0.05	± 0.02
	*p* value	0.361	0.721	0.362	0.837	0.890	0.960	0.872	0.717	0.036	0.023	0.892	0.008
Age of hens (weeks)	I (19)	18.25^d^	12.74^b^	31.14^c^	9.34^d^	41.37^a^	28.38^b^	69.75^a^	20.94^d^	12.01^a^	110.30^a^	1.064	1.029
	II (24)	20.13^c^	15.27^a^	35.39^bc^	11.74^c^	38.82^b^	29.29^a^	68.11^b^	22.87^d^	9.92^b^	100.89^b^	1.058	1.031
	III (29)	21.15^b^	14.94^b^	36.08^bc^	12.51^c^	39.79^ab^	27.80^b^	67.58^c^	23.30^c^	8.89^c^	95.52^c^	1.050	1.029
	IV (34)	21.08^b^	15.71^a^	36.80^b^	14.43^b^	37.35^c^	27.81^b^	65.16^d^	25.65^b^	8.49^c^	92.70^c^	1.044	1.037
	V (39)	24.68^a^	12.85^b^	37.53^a^	16.69^a^	41.41^a^	21.48^b^	62.88^d^	28.00^a^	8.85^c^	93.67^c^	1.025	1.029
	SD	2.72	3.25	3.41	1.18	4.75	5.32	2.42	2.12	1.68	7.68	0.04	0.02
	*p* value	0.000	0.000	0.000	0.000	0.000	0.000	0.000	0.000	0.000	0.000	0.000	0.290
	Interaction	0.215	0.716	0.498	0.763	0.440	0.416	0.496	0.610	0.351	0.383	0.021	0.012

Different letters (a, b) indicate a significant difference between treatments (A–B) and periods (I–V) at *p* < 0.05.

*Standard deviation.

^1^TREATMENT: A, control with soybean meal; B, experimental with yellow lupin, narrow-leaved lupin and peas.

**Table 4:** Mean values (x̅) and standard deviations (± SD) for egg yolk colour.

Factor	Indicator	Yolk colour (score)	Colour		
			L*	a*	b*
Group	A	5.40^b^	51.74	4.94^a^	26.87
	B	5.85^a^	51.34	3.95^b^	20.93
	± SD*	± 1.50	± 3.78	± 2.20	± 6.96
	*p* value	0.009	0.069	0.000	0.135
Age of hens (weeks)	I (19)	5.42^b^	47.92^c^	1.59^d^	18.84^d^
	II (24)	6.24^a^	47.45^c^	3.52^c^	21.32^c^
	III (29)	5.78^ab^	54.84^a^	4.99^b^	30.98^b^
	IV (34)	5.58^ab^	54.55^a^	5.92^b^	32.93^a^
	V (39)	5.12^b^	52.89^b^	6.16^a^	31.86^ab^
	±SD*	1.39	1.99	1.35	3.37
	*p* value	0.000	0.000	0.000	0.000
	Interaction	0.027	0.117	0.000	0.001

Different letters (a, b) indicate a significant difference between treatments (A–B) and periods (I–V) at *p* < 0.05.

*Standard deviation.

^1^TREATMENT: A, control with soybean meal; B, experimental with yellow lupin, narrow-leaved lupin and peas.

Table 6 presents results from the analysis of fatty acids composition and the content of saturated, monounsaturated and polyunsaturated FA in yolk lipids. Significantly lower content of C15:0 and C18:1n9, and significantly higher content of C18:2n6 and C18:3n3 were found in group B (*p* < 0.05) compared to group A. The content of monounsaturated fatty acids (MUFA) and OMEGA 9 FA was significantly lower in group B (*p* = 0.020, *p* = 0.018, respectively). However, the content of polyunsaturated FA (PUFA), OMEGA 3 and 6, the PUFA/SFA ratio, OMEGA 9/6 and OMEGA 9/3 were significantly higher in group B (*p* < 0.05). Diet had no significant effect on other parameters presented in Tables 1, 2, 3, 4, 5, 6 (*p* < 0.05).

**Table 5:** Mean values (x̅) and coefficients of variation (V) for the content and enzymatic activity of lysozyme in albumen.

Factor		Concentration of lysozyme (%) in albumen		Lysozyme activity (U/mg) in albumen	
		Thick	Thin	Thick	Thin
Group^1^	A	0.219	0.433	46,641	92,089
	B	0.211	0.444	44,746	94,271
	V* (%)	23.18	11.69	23.18	11.69
	p value	0.285	0.296	0.285	0.296
Age of hens (weeks)	I (19)	0.187^cd^	0.434^ab^	39730^cd^	92254^ab^
	II (24)	0.242^ab^	0.455^ab^	51524^ab^	96720^ab^
	III (29)	0.173^d^	0.426^ab^	36840^d^	90466^ab^
	IV (34)	0.210^bc^	0.459^a^	44707^bc^	97621^a^
	V (39)	0.264^a^	0.415^b^	56112^a^	88252^b^
	V* (%)	17.48	10.89	17.48	10.89
	*p* value	0.000	0.011	0.000	0.011
	Interaction	0.259	0.000	0.259	0.000

Different letters (a, b) indicate a significant difference between treatments (A–B) and periods (I–V) at p < 0.05.

*Coefficients of variation.

^1^TREATMENT: A, control with soybean meal; B, experimental with yellow lupin, narrow-leaved lupin and peas.

**Table 6:** Fatty acids composition (%) in yolk lipids (mean values x̅ and standard deviation ± SD) and the content of saturated, monounsaturated, and polyunsaturated fatty acids (%) in yolk lipids (x̅ ± SD).

Fatty acids	Group^1^		± SD*	Age of hens (weeks)			p value		
	A	B		I (19)	II (29)	III (39)	Group	Age of hens	Interaction
**Content of fatty acids (%) in yolk lipids**									
C14:0	0.49	0.48	± 0.06	0.48	0.50	0.47	0.695	0.601	0.449
C15:0	0.10^a^	0.11^b^	± 0.02	0.10	0.10	0.11	0.047	0.244	0.576
C16:0	46.77	46.36	± 1.54	47.45^a^	45.51^b^	46.75^ab^	0.423	0.014	0.773
C16:1	1.90	1.85	± 0.35	2.13^a^	1.95^b^	1.53^b^	0.586	0.000	0.612
C17:0	0.24	0.26	± 0.05	0.24	0.24	0.28	0.172	0.121	0.477
C18:0	16.64	17.15	± 1.07	16.69	16.97	17.04	0.226	0.768	0.649
C18:1n9	21.81^a^	20.76^b^	± 1.35	20.64^b^	22.49^a^	20.72^b^	0.015	0.001	0.823
C18:2n6	9.52^b^	10.34^a^	± 0.95	9.53	9.78	10.49	0.021	0.066	0.674
C18:3n3	0.34^b^	0.38^a^	± 0.05	0.35	0.38	0.35	0.034	0.511	0.417
C20:1n9	0.10	0.11	± 0.02	0.10^b^	0.12^a^	0.10^b^	0.431	0.009	0.691
C20:2n6	0.15	0.17	± 0.04	0.18^a^	0.15^ab^	0.14^b^	0.078	0.019	0.894
C22:0	1.72	1.79	± 0.25	1.86	1.62	1.78	0.430	0.078	0.334
C24:0	0.22	0.25	± 0.05	0.26^a^	0.21^b^	0.24^ab^	0.158	0.035	0.436
**Content of saturated, monounsaturated, and polyunsaturated fatty acids (%) in yolk lipids**									
SFA	66.18	66.40	± 0.90	67.07^a^	65.13^b^	66.67^a^	0.527	0.000	0.322
UFA	33.81	33.60	± 0.89	32.93^b^	34.87^a^	33.33^b^	0.545	0.000	0.328
MUFA	23.81^a^	22.71^b^	± 1.18	22.87^b^	24.56^a^	22.35^b^	0.020	0.001	0.744
PUFA	10.01^b^	10.89^a^	± 0.92	10.06	10.31	10.98	0.017	0.094	0.639
OMEGA 3	0.34^b^	0.38^a^	± 0.05	0.35	0.38	0.35	0.034	0.511	0.417
OMEGA 6	9.67^b^	10.51^a^	± 0.95	9.71	9.93	10.63	0.021	0.066	0.674
OMEGA 9	21.91^a^	20.87^b^	± 1.09	20.74^b^	22.61^a^	20.82^b^	0.018	0.010	0.823
DFA	50.46	50.76	± 1.17	49.63^b^	51.84^a^	50.37^b^	0.512	0.002	0.766
OFA	47.26	46.84	± 1.32	47.92^a^	46.00^b^	47.22^a^	0.421	0.018	0.808
UFA/SFA	0.51	0.51	± 0.02	0.49^b^	0.54^a^	0.50^b^	0.535	0.000	0.321
MUFA/SFA	0.36	0.34	± 0.02	0.34^b^	0.38^a^	0.34^b^	0.035	0.000	0.616
PUFA/SFA	0.15^b^	0.16^a^	± 0.02	0.15	0.16	0.16	0.025	0.101	0.576
DFA/SFA	0.76	0.76	± 0.03	0.74^b^	0.80^a^	0.76^b^	0.850	0.000	0.565
DFA/OFA	1.07	1.09	± 0.06	1.04^b^	1.13^a^	1.07^ab^	0.472	0.007	0.830
OMEGA 6/3	27.77	29.28	± 2.75	30.63^a^	26.91^b^	28.03^ab^	0.179	0.028	0.325
OMEGA 9/6	2.00^b^	2.29^a^	± 0.28	1.99	2.30	2.16	0.011	0.068	0.910
OMEGA 9/3	55.46^b^	67.18^a^	± 10.87	61.67	61.68	60.61	0.012	0.979	0.478

Different letters (a, b) indicate a significant difference between treatments (A–B) and periods (I–V) at p < 0.05.

*Standard deviation.

^1^TREATMENT: A, control with soybean meal; B, experimental with yellow lupin, narrow-leaved lupin and peas. C14:0, myristic acid; C15:0, pentadecanoic acid; C16:0, palmitic acid; C16:1, palmitoleic acid; C17:0, margaric acid; C18:0, stearic acid; C18:1n9, oleic acid; C18:2n6, linoleic acid; C18:3n3, alpha-linolenic acid; C20:1n9, eicosanoic acid; C20:2n6, eicosadienoic acid; C22:0, behenic acid; C24:0, lignoceric acid; SFA, saturated fatty acid; UFA, unsaturated fatty acid; MUFA, monounsaturated fatty acid; PUFA, polyunsaturated fatty acid; OMEGA 3,6,9, unsaturated fatty acid from PUFA (n-3, n-6) and MUFA (n-9); DFA, hypocholesterolemic acid; OFA, hypercholesterolemic acid.

### Effect of age of hens on the egg quality

Egg weight and egg surface area were significantly higher in eggs produced on date V than on other dates, while the egg shape index for eggs produced on date II was significantly higher compared to dates III and V (*p* = 0.000) (Table 1). The analysis of data in Table 2 revealed that eggs produced on dates IV and V had whiter, thicker and denser shells (*p* < 0.05). Eggshell strength was significantly lower on date I compared to other dates (*p* = 0.000), while eggshell weight was significantly higher on date V (*p* = 0.000). Considering the quality of egg components (Table 3), the weight of thick albumen, total albumen and yolk weight, as well as the proportion of yolk in the egg were significantly higher on date V (*p* = 0.000, for all listed traits). Eggs produced at the beginning of the study period (date I) were characterised by a higher proportion of total albumen, height of thick albumen and higher Haugh score (HU) (*p* = 0.000, for all listed traits). The proportion of thick albumen was higher on dates I and V compared to dates II and IV (*p* = 0.000), and on date II eggs were characterised by a higher proportion of thin albumen, and the weight of thin albumen was higher on dates II and IV compared to other hens age (*p* = 0.000). Yolk colour assessed by DSM was significantly darker on date II than on dates I and V (*p* = 0.000). Colour saturation expressed by lightness (L*) was higher on dates III and IV than on other dates, redness (a*) was higher on date V than on dates I–IV, and yellowness was higher on date IV than on dates I–III (*p* = 0.000) (Table 4). There were significant differences in the content and activity of lysozyme (Table 5) between eggs produced on date V and those produced on dates I, III and IV, and the content of lysozyme in thin albumen from eggs produced on date IV was higher than in eggs produced on date V (*p* < 0.05). Lysozyme activity in thin albumen was significantly higher on date IV than on date V (*p* = 0.011).

Fatty acids composition and the content of SFA, MUFA and PUFA were analysed on three dates of hens age (I–III, Table 6). Significantly higher content was found for C16:0, C16:1, C20:2n6 and C24:0 on date I, and for C18:1n9 and C20:1n9 on date II, while on date III the content of these fatty acids was significantly lower or similar to that on other dates (*p* < 0.05). The content of SFA and OFA (hypercholesterolemic fatty acids) was significantly higher on dates I and III (*p* = 0.000), while for UFA (unsaturated fatty acids) and MUFA, OMEGA 9, DFA, UFA/SFA, MUFA/SFA and DFA (hypocholesterolemic fatty acids)/SFA the analysis revealed significantly lower levels (*p* < 0.05) than on date II. The DFA/OFA ratio was significantly higher on date II, while the OMEGA 6/3 ratio was lower compared to date I (*p* < 0.05). There were no significant differences in results presented in Tables 4, 5, 6, 7, 8, 9 between age of hens.

**Table 7:** Composition of concentrates for Rosa 1 hens.

Component (%)	A^a^	B^b^
Maize	28.000	6.800
Soybean meal (Hipro)	41.697	–
Peas (Muza)	–	11.000
Yellow lupin (Mister)	–	25.000
Narrow-leaved lupin (Boruta)	–	22.000
Rapeseed oil	4.000	7.000
Monocalcium phosphate	2.500	3.000
Limestone	19.000	19.000
Fodder salt	0.300	0.300
Sodium carbonate	0.600	0.800
l-lysine /technically pure/	0.200	0.600
dl-methionine	0.300	0.500
l-threonine	0.100	0.400
l-tryptophane /technically pure/	0.003	0.100
l-Valine	0.300	0.500
Premix for laying hens 1%^c^	3.000	3.000

^a^A, group fed with soybean meal (var. Hipro).

^b^B, group fed with legume seeds.

^c^The vitamin and mineral premix provides per kg of diet: Cu, 10 mg; Fe, 60 mg; Mn, 80 mg; Zn, 60 mg; I, 1.5 mg; Se, 0.3 mg; vitamin A, 10.000 IU; vitamin D, 2500 IU; vitamin E, 25 IU; vitamin K, 1.0 mg; vitamin B1, 2.0 mg; vitamin B2, 8.0 mg; vitamin B6, 2.5 mg; vitamin B12, 0.01 mg; vitamin PP (nicotinamide pancreatic polypeptide), 30.0 mg; vitamin B5, 15.0 mg; vitamin B9, 0.5 mg; and biotin, 0.15 mg.

**Table 8:** Calculated nutritional value of feed mixture (55% wheat and 45% concentrate).

Parameter	Nutritional value
Metabolizable energy, MJ/kg	11.30
Crude protein, %	16.20
Calcium, %	3.50
P—available, %	0.39
Lysine, dig., %	0.75
Methionine + cystine, dig., %	0.63
Thyrosine, dig., %	0.16
Threonine, dig., %	0.53
Valine, dig., %	0.68

**Table 9:** Chemical composition of narrow-leaved lupin seeds, var. Boruta, yellow lupin, var. Mister, and peas, var. Muza used in feed mixture.

Ingredients	Content		
	Narrow-leaved lupin, Boruta	Yellow lupin, Mister	Pea, Muza
Dry weight, %	88.62	89.01	86.65
Crude ash, %	3.78	4.15	3.14
Crude protein, %	36.88	38.98	27.57
Crude fibre, %	15.09	19.23	6.34
ADF, %	21.43	24.24	7.97
NDF, %	25.92	28.24	13.88
Crude fat, %	5.81	5.26	1.32
Starch, %	–	–	44.23
Energy, MJ/kg	20.73	20.49	19.45
kcal/kg	4951.28	4893.95	4645.55
Viscosity, cP	1.21	1.09	1.29
Asp, %	8.91	8.81	10.49
Thr, %	3.15	3.17	3.54
Ser, %	4.11	4.24	4.38
Glu, %	23.77	24.46	19.46
Pro, %	6.52	6.08	5.77
Gly, %	4.01	3.47	3.83
Ala, %	3.33	2.83	3.81
Val, %	3.72	3.17	4.35
Iso, %	3.68	3.20	3.66
Leu, %	6.64	6.50	6.63
Tyr, %	3.07	3.24	3.26
Phe, %	3.46	4.24	5.00
His, %	2.91	3.32	3.37
Lys, %	4.49	4.76	6.52
Arg, %	11.65	10.12	8.82
Total amino acids, %	39.39	39.29	42.53
Ca, g/kg d.w.	3.33	2.95	1.27
K, g/kg d.w.	13.45	12.66	12.72
P, g/kg d.w.	6.84	7.47	5.10
Na, g/kg d.w.	0.08	0.08	0.062
Mg, g/kg d.w.	2.10	3.14	1.47
Mn, g/kg d.w.	0.13	0.08	0.02
Cu, g/kg d.w.	0.04	0.02	0.02
Fe, g/kg d.w.	0.07	0.13	0.07
Zn, g/kg d.w.	0.07	0.07	0.06
Total alkaloids, mg/kg	440	270	–
Angustifoline, %	12.45	–	–
Isolupanine, %	4.56	–	–
Lupanine, %	56.17	–	–
130H lupanine, %	26.72	–	–
Sparteine, %	–	33.60	–
Lupinine, %	–	63.29	–
Oligosaccharides, g/kg d.w.	8.77	8.56	8.34
Raffinose, g/kg d.w.	1.20	1.10	0.90
Stachyose, g/kg d.w.	5.61	4.94	3.86
Verbascose, g/kg d.w.	1.96	2.53	3.59
P-phytate, %	0.42	0.70	0.44

ADF, acid detergent fiber; NDF, neutral detergent fiber; Asp, aspartic acid; Thr, threonine; Ser, serine; Glu, glutamic acid; Pro, proline; Gly, glycine; Ala, alanine; Val, valine; Iso, isoleucine; Leu, leucine; Tyr, tyrosine; Phe, phenylalanine; His, histidine; Lys, lysine; Arg, arginine; Ca, calcium; K, potassium; P, phosphorus Na, sodium; Mg, magnesium; Mn, manganese; Cu, copper; Fe, iron; Zn, zinc.

### Interaction between experimental factors

A significant interaction between grouping variables (diet and age of hens) was found for shell strength (*p* = 0.036, Table 2), density of thick albumen (*p* = 0.021, Table 3), yolk colour measured by DSM, colour parameters a* and b* (*p* = 0.027; 0.000; 0.001; Table 4) and the content and activity of lysozyme in thin albumen (*p* = 0.000, Table 5).

## Discussion

As mentioned in Materials and Methods, a parallel study was conducted under the project to investigate the quality of eggs from Hy-Line Brown hens managed in a semi-intensive system and fed a diet with the inclusion of yellow lupin seeds (10–25%) and peas (10%)^18^. The authors reported that a 10–20% inclusion of lupin seeds with a 10% inclusion of peas had no negative effect on most egg traits and had a positive effect on the content of omega fatty acids and yolk pigmentation. However, the highest dose of narrow-leaved lupin (25%) in the feed ration was associated with reduced weight of eggs.

In the studies by Rutkowski et al*.*^19^, feed concentrates with narrow-leaved lupin, yellow lupin and peas were used in feed for laying hens. It was found that the presence of anti-nutritional substances in legume seeds could reduce the production results, with a beneficial increase in the color of the yolk, protein index and HU score. However, the proportion of these seeds at the level of 19.48% could be accepted as a substitute for soybean meal. Other studies have demonstrated that the inclusion of yellow lupin in feed had no significant effect on the parameters of egg quality. In addition, a 15% inclusion of lupin was associated with a higher proportion (%) of thin albumen in eggs, and a 10–25% inclusion of lupin reduced the proportion of eggshell in each nutritional group^11^. Our study did not reveal any significant differences in these quality traits. A diet based on unprocessed peas had no significant effect on the quality of eggs from laying hens^20^. Laudadio and Tufarelli^10^ reported that the inclusion of lupin in the diet of laying hens only resulted in a stronger pigmentation of yolk (a scale of 1–15), which could be associated with the amount of natural pigments in lupins. Other researchers found that the inclusion of peas improved the colour of yolk^21^. Our study revealed a stronger pigmentation of yolk colour in the treatment group, which is consistent with findings by other authors^11,18,20,21^. Yolk redness (a*) in the control group was significantly higher, but it is not entirely clear whether this could be attributed to the diet, which was discussed earlier by Sasyte et al*.*^22^. The colour of the yolk depends to a large extent on carotenoids contained in plant seeds, which are responsible for the yellow or red colour^17^. Depending on the variety of lupins, the proportion of carotenoids varies, with lutein and beta-carotene being present in both yellow and narrow-leaved lupine seeds^23^, which may explain the results of the discussed studies on the colour of yolk. Similarly, pea seeds containing xanthophylls could have an effect on the colour of the egg yolk^20^.The diet with the inclusion of lupin and peas used in our study had a positive effect on the height of thick albumen and the Haugh score. The quality of albumen is reflected in Haugh units, which is expressed as a ratio of the thick albumen height and egg weight, whereas the higher Haugh scores indicate a better quality of albumen^24,25^. However, the amount of thick albumen and Haugh units, as indicators of egg quality and freshness, depend on the origin, age or nutrition, mainly on the laying date and storage time^26^. These features mainly depend on changes in the protein gel structure, which is influenced by the content of ovalbumin and ovomucin, which influence the lysozyme content and its activity. During the storage time, lysozyme level decreasing^26–28^. Nevertheless, Roberts^14^ indicates that the share of protein in hen’s nutrition affects the quality of protein, expressed in Haugh units. In our study we found a beneficial composition of fatty acids, including a significantly higher content of OMEGA 3 and 6, as well as α-linolenic fatty acid. Yellow lupin, as a feed material in an experimental feed, is characterized by a high content of OMEGA 6 acids, including LA (C18: 2n-6) and C20: 2n-6 (eicosadienoic acid)^29^. Similar findings were reported from a study in which laying hens received a diet based on yellow lupin^29^. Researchers demonstrated that lupin seeds at a dose of 300 g/kg increased the content of the above-mentioned fatty acids in egg yolk lipids, which was considered a beneficial effect. To sum up, the beneficial composition of fatty acids in eggs from laying hens fed a diet based on yellow lupin was attributed to the inclusion of lupin seeds, because the cited authors^29^ found higher content of OMEGA 6 in yellow lupin compared to white and blue lupin. Drażbo et al*.*^30^ also reported that a 20% inclusion of blue lupin seeds in the diet of laying hens had a positive effect on egg quality, including fatty acids composition in yolk lipids.

The age of laying hens influenced many parameters of eggs analysed in our study. Zita et al*.*^31^ reported that the weight of eggs, yolk and the proportion of yolk in eggs, as well as the Haugh score increased with the age of birds. However, older hens produced eggs with a lower proportion of albumen and shell, but with thicker and stronger shells. Similar observations were made in our study, except for the improved quality of albumen (HU). Kraus and Zita^32^ concluded that the age of hens is one of the most important factors influencing egg quality traits. Consistent with our study, Kraus et al*.*^33^ reported that the age of hens influenced all quality traits of eggs.

In conclusion, the proposed balanced diet based on yellow lupin (var. Mister), narrow-leaved lupin (var. Boruta) and peas (var. Muza) had no negative effect on the quality of eggs. The study demonstrated an improved egg shape index, yolk colour and albumen quality measured in Haugh units. Importantly, the alternative diet had a beneficial effect on fatty acids composition in egg yolk lipids, which should encourage consumers to purchase such eggs. The proposed feed could be an interesting alternative for egg producers operating small farms, who have limited, if any, opportunities to produce soybean meal (SBM). The use of alternative feed components will allow for partial self-sufficiency in the production of feed rich in protein and will reduce the use of feeds from genetically modified plants, such as SBM, which is important from the consumer perspective. An important element that should be taken into account when composing the ration is anti-nutritional substances that can potentially limit the use of legumes in the feeding of laying hens. The age of laying hens influenced all parameters of egg quality, which may be associated with the normal physiology of egg laying.

## Materials and methods

The present study was a part of the research project [Resolution No. 222/2015], which aimed at investigating the effect of legume seeds as protein-rich feed components alternative to soybean meal used in the diet of laying hens. In parallel to the present study, experiments were conducted on Hy-Line Brown laying hens managed in an intensive (cage) system. In these two parallel studies same analytical methods were used, and they were presented in a publication by Kowalska et al*.*^18^.

The study concerned the analysis of physicochemical characteristics of eggs obtained from hens managed on a small commercial farm. Therefore, according to Directive no. 2010/63/EU, the study did not require approval from a Local Ethics Committee. No approval was required under Resolution no. 13/2016 of the National Ethics Committee for Animal Experiments of 17 June 2016.

### Bird management

Rosa 1 laying hens were managed at a small farm. The hens used in the test are three-stemmed hybrids from Rhode Island Red (paternal component)—R55 and two Sussex lines (maternal component)—S11 and S55. They are commonly kept hens for the production of consumption eggs. Hens were divided into two groups: control (A), fed a diet based on soybean meal (SBM), and the treatment group (B), fed a diet with the inclusion of narrow-leaved lupin, var. Boruta (*Lupinus angustifolius* L.), yellow lupin, var. Mister (*Lupinus luteus* L.) and peas, var. Muza (*Pisum sativum* L.). Detailed compositions of feed concentrates, and nutritional values of feeds are presented in Tables 7 and 8. Table 9 shows the chemical composition of seeds from narrow-leaved lupin, yellow lupin and peas used in feed mixtures for laying hens. The chemical composition of legume seeds was analysed using methodology previously described by Biesek et al*.*^34^.

Feed for the control group and treatment group contained 45% of protein-rich concentrate and 55% of wheat. Hens were managed in a semi-intensive barn system and the floor in the hen house was covered with chopped wheat straw. Hens had access to pens located directly behind the hen house, and each group used a separate pen. The research was done in the commercial environments, therefor hens from each group had common pens. The study’ aim was the egg quality analysis, no production performance. It is explained, due to the possibilities of statistical analysis of eggs traits. Each egg was individual unit. The temperature in the hen house was maintained at 15–16 °C, and the birds were exposed to a standard photoperiod of 16 h of light and 8 h of darkness. Environmental conditions were consistent with standards for the management of hens during the egg production period. Birds received feed and fresh drinking water ad libitum.

### Egg collection

Three hundred hen eggs were collected on five dates (I–V; from week 2 to week 22 of egg production) and used for the analysis of egg components and shells. In week 2 of egg production hens were 19 weeks old. Physicochemical parameters of eggs were analysed at 4-week intervals within 24 h after egg collection (30 eggs from each group on each date). Eggs were randomly selected. We analysed egg components, the proportion of egg components in the total weight of egg, albumen quality, the content and activity of lysozyme in thick and thin albumen, yolk colour and eggshell porosity. Fatty acids composition was analysed in weeks 2 (I), 12 (II) and 22 (III) of egg production for 10 eggs collected on each date.

### Egg quality

Thirty eggs were collected from each group for analysis. Each egg was regarded as a sample, and the mean for each group was calculated based on measurements. Eggs were weighed (RADWAG scales, PS 750/X, accuracy of 0.01 g), the egg shape index (%) was calculated as the ratio of the height to the width of eggs (Mitutoyo digital calliper, Quantu Mike), and the egg surface area (cm^2^) was calculated from the formula P_s_ = 4.835 × W^0.662^, where W—egg weight (g)^35^. Eggshell strength was measured on an egg crusher (kg/cm^3^, Egg Force Reader, Orka Food Technology Ltd.). Egg shell whiteness was analysed (QCR reflectometer, TSS). The height of thick albumen was measured using a QCD apparatus (TSS). Yolk colour was estimated by means of a subjective and an objective method. The subjective method relied on a 1–15 La Roche scale, and the objective method relied on colorimetric analysis (Konica Minolta) and the CIE Lab system (where L* is lightness, a* is redness, and b* is yellowness)^36^. The Haugh score was calculated from the formula HU = 100 lg (H + 7.7 – 1/7W^0.37^), (H—height of thick albumen (mm), W—egg weight (g)), following Williams^37^. The specific density of thick albumen and yolk was determined using KIT-128 for the analysis of density of liquids and solids, and RADWAG 750/X scales. Collected shells were dried in an oven (SUB 100 M) at 105 °C for 3 h and measured for thickness (a screw thread micrometer, TSS). A 2–3 g sample was prepared from each shell (the equatorial region) and used for the measurement of density (KIT-128, RADWAG). Distilled water (22 °C) was used as a reference liquid in the analysis of density of liquids and solids. Values obtained from the measurement of egg weight and the weight of egg components (yolk, albumen, shell) were used to calculate the proportion of these components in eggs.

### Lysozyme and its enzymatic activity in albumen

The methodology for the analysis of the content of lysozyme and its activity in albumen was described by Adamski et al*.*^38^. Thick and thin albumen (10 samples from each group) were stored in containers and used for analysis. The concentration (%) of lysozyme and its hydrolytic activity (U) were measured spectrophotometrically (SP-830 plus, Metertech). It was assumed that one unit of lysozyme would produce a ΔA450 of 0.001 per minute at pH 6.24 using a suspension of *Micrococcus lysodeikticus* as the substrate in a 2.6 ml reaction mixture (2.5 ml of bacterial suspension and 0.1 ml of lysozyme in a cuvette of 1 cm path length). Decrease in the absorbance of the analysed solution was calculated from the formula: ΔA = A_t0_ – A_t_ (U/min) (ΔA, decrease in absorbance of solution; A_t0_, absorbance of bacterial suspension at *t*_*0*_ time; At, absorbance of bacterial suspension after *t* time.

### Composition of fatty acids in egg yolk lipids

The composition of fatty acids in egg yolk lipids was analysed at the beginning, at the peak, and at the end of the egg production period (weeks 19, 20, 39 age of hens). Five yolks from each group were used for analysis. Fat was extracted from yolks^39^, and fatty acid methyl esters were identified using the PN-EN ISO 12966-2 standard^40^. Yolks were lyophilized (Alpha plus, Donserv), fat was extracted, and samples were filtered and evaporated. According to the above-mentioned standard, fat was dissolved in isooctane and transmethylated with potassium hydroxide solution. Next, potassium hydroxide was neutralized with acidic sodium sulfate and esters were salted out with sodium chloride. The prepared esters were analyzed on a gas chromatograph (Agilent Technologies, type 7890 B). Fatty acid methyl esters were identified using the Supelco 37 standard FAME Mix component. Analytical parameters were presented in a publication by Kowalska et al*.*^18^.

### Statistical analysis

Gathered data were analysed with statistical software^41^. Means for each analysed trait were calculated for nutritional groups and age of hens. Standard deviation (± SD) and coefficient of variation (v) were calculated. The two-way ANOVA model was used to analyse variance. Differences in the values of the examined traits for each grouping variable (diet and age of hens) were calculated. The significance of differences was verified using the post-hoc Tukey test. Analysis was performed using the one-way ANOVA model with consideration of the effects of subclasses. Data were verified for the interaction between variables (diet × age of hens) for each analysed trait. Differences were considered significant at p < 0.05.

### Ethics

The study concerned the analysis of physicochemical characteristics of eggs obtained from hens managed on a small commercial farm. Therefore, according to Directive no. 2010/63/EU, the study did not require approval from a Local Ethics Committee. No approval was required under Resolution no. 13/2016 of the National Ethics Committee for Animal Experiments of 17 June 2016.
